# From Adipose Tissue to Cardiac Repair: Extracellular Vesicles in Cardiovascular Regeneration

**DOI:** 10.1111/jcmm.71148

**Published:** 2026-04-29

**Authors:** Karolina Ignaczak, Pola Ochocka, Karol Sadowski, Weronika Ploch, Dominika Tomkiel, Mariusz Tomaniak, Wioletta Olejarz

**Affiliations:** ^1^ First Chair and Department of Cardiology Medical University of Warsaw Warsaw Poland; ^2^ InterCinic Group and Laboratory Medical University of Warsaw Warsaw Poland; ^3^ CardioQuant Group and Laboratory Medical University of Warsaw Warsaw Poland; ^4^ Department of Biochemistry and Pharmacogenomics Medical University of Warsaw Warsaw Poland; ^5^ Centre for Preclinical Research Medical University of Warsaw Warsaw Poland

**Keywords:** adipose tissue, adipose‐derived extracellular vesicles, adipose‐derived stem cells, cardiovascular diseases, extracellular vesicles

## Abstract

Adipose‐derived extracellular vesicles (ADEVs) secreted by adipose tissue have gained increasing attention due to their regenerative and immunomodulatory properties in cardiovascular diseases. They carry proteins, lipids, and nucleic acids that mediate intercellular communication and influence cardiac pathophysiology. In atherosclerosis, ADEVs promote vascular regeneration, protect endothelial integrity, modulate macrophage polarization, foam cell formation, and regulate lipid homeostasis. In myocardial infarction, they limit apoptosis, reduce fibrosis, stimulate angiogenesis, and regulate inflammation through miRNA‐dependent paracrine mechanisms. While preclinical studies support their therapeutic relevance, clinical translation remains at an early stage due to fragmented evidence and lack of standardization in isolation methods, dosing, and safety assessment. This review synthesizes current pathophysiological and translational evidence on the role of ADEVs in cardiovascular repair and provides a structured perspective to guide the development of ADEV‐based therapies toward clinical application.

**Abbreviations**
ADEVs: adipose‐derived extracellular vesicles
ADMSCs: adipose‐derived mesenchymal stem cells
ADSCs: adipose‐derived stem cells
AMI: acute myocardial infarction
CABG: coronary artery bypass grafting
CAD: coronary artery disease
EVs: extracellular vesicles
MI: myocardial infarction

## 1 Introduction

Cardiovascular diseases remain the leading cause of mortality worldwide despite major advances in interventional procedures and pharmacotherapy [1, 2, 3]. Many patients still suffer from ischemic symptoms or fail to fully recover even when treated according to guidelines. This highlights a therapeutic gap and signals the need for novel, biology‐driven strategies [4, 5]. Traditional cell‐based therapies have shown promise, yet their clinical use is limited by safety concerns, poor engraftment, and difficulties in standardization [6, 7]. In this context, cell‐free approaches such as extracellular vesicles (EVs) are particularly attractive. EVs act as natural mediators of intercellular communication, capable of transferring bioactive cargo and reprogramming target cells relevant to cardiac injury and repair, while avoiding limitations of cell transplantation [8, 9, 10, 11, 12, 13].

Within this field, adipose‐derived extracellular vesicles (ADEVs) are particularly promising. Adipose tissue is abundant, easily accessible, and functions as a systemic endocrine organ. Consequently, ADEVs represent a major fraction of circulating EVs and form a physiological adipo‐cardiac signalling axis [14, 15, 16, 17]. These features distinguish ADEVs from other EV populations and make them especially relevant for cardiovascular disease.

Emerging preclinical evidence suggests that ADEVs have the capacity to modulate vascular inflammation, lipid metabolism, and myocardial repair. Nonetheless, ADEV‐based strategies remain confined to the preclinical domain, whereas clinical research has thus far predominantly focused on adipose‐derived stem cells, with heterogeneous outcomes regarding both efficacy and safety [18, 19, 20, 21, 22, 23, 24, 25]. Translation will require clarification of which ADEV cargo components drive therapeutic benefit, optimization of isolation and dosing strategies, and the establishment of reproducible delivery methods. Equally important is the systematic evaluation of safety, since ADEVs derived from diseased, obese, or tumour‐associated tissue may be harmful [5, 8, 13, 14, 26]. Finally, adherence to methodological standards such as MISEV2023 will be essential to ensure reproducibility and comparability across studies [27].

This review provides a summary of current knowledge about the ADEVs from both preclinical and clinical perspectives. The first part of the paper focuses on in vitro findings regarding ADEVs, highlighting the mechanisms of their anti‐atherosclerotic and pro‐regenerative properties. Subsequently, clinical trials investigating the use of adipose‐derived stem cells (ADSCs) in cardiovascular diseases are discussed, with a particular focus on the pivotal trends in trial design and the reported efficacy and safety outcomes. In the following section, potential harmful effects and safety considerations are discussed, with emphasis on factors that should guide future translational studies. Finally, we provide an outlook on upcoming trials in the domain of ADSCs.

## 2 Description of Adipose‐Derived Extracellular Vesicles

ADEVs are nanosized lipid bilayer vesicles secreted by adipocytes and ADSCs [14, 15, 16]. In contrast to cell‐based therapies, ADEVs do not elicit a significant immunological response due to their acellular nature. Furthermore, compared with stem cells, ADEVs avoid the risk of uncontrolled proliferation and remain stable in circulation, making them a promising and safe cell‐free alternative to ADSC‐based therapies [10, 11, 13, 28, 29, 30, 31].

Like other extracellular vesicles, ADEVs contain a set of conserved proteins widely recognized as common EV markers, including tetraspanins (CD9, CD63, CD81), heat shock proteins (HSP70, HSP90), and ESCRT‐related molecules such as TSG101 and Alix [27, 32, 33]. Beyond these general markers, ADEVs carry proteins reflecting the metabolic and endocrine functions of adipose tissue, including adiponectin, perilipin A, angiogenesis‐related proteins (VEGF, angiopoietin‐like proteins), and extracellular matrix remodelers such as MMP2 and MMP9 [12, 16, 34, 35, 36].

In terms of RNA content, ADEVs are enriched in miRNAs involved in adipogenesis, vascular homeostasis, and inflammation—including miR‐21, miR‐126, miR‐146a, and miR‐155—which influence endothelial repair, macrophage polarization, and inflammatory signalling [14, 36, 37, 38, 39]. Compared with EVs secreted by other adult stem cells, ADEVs display a distinct transcriptomic profile [13, 14]. For example, bone marrow mesenchymal stem cells‐derived EVs (MSC‐EVs) are enriched in osteogenesis and haematopoiesis‐related miRNAs (e.g., miR‐196a, miR‐223) [40, 41], while umbilical cord‐derived EVs contain angiogenic and immunomodulatory miRNAs [42]. This highlights that ADEVs are particularly suited to modulate metabolic and inflammatory pathways, while retaining regenerative potential observed across other stem cell‐derived EVs [13].

## 3 The Anti‐Atherosclerotic Potential of ADEVs

**FIGURE 1:**
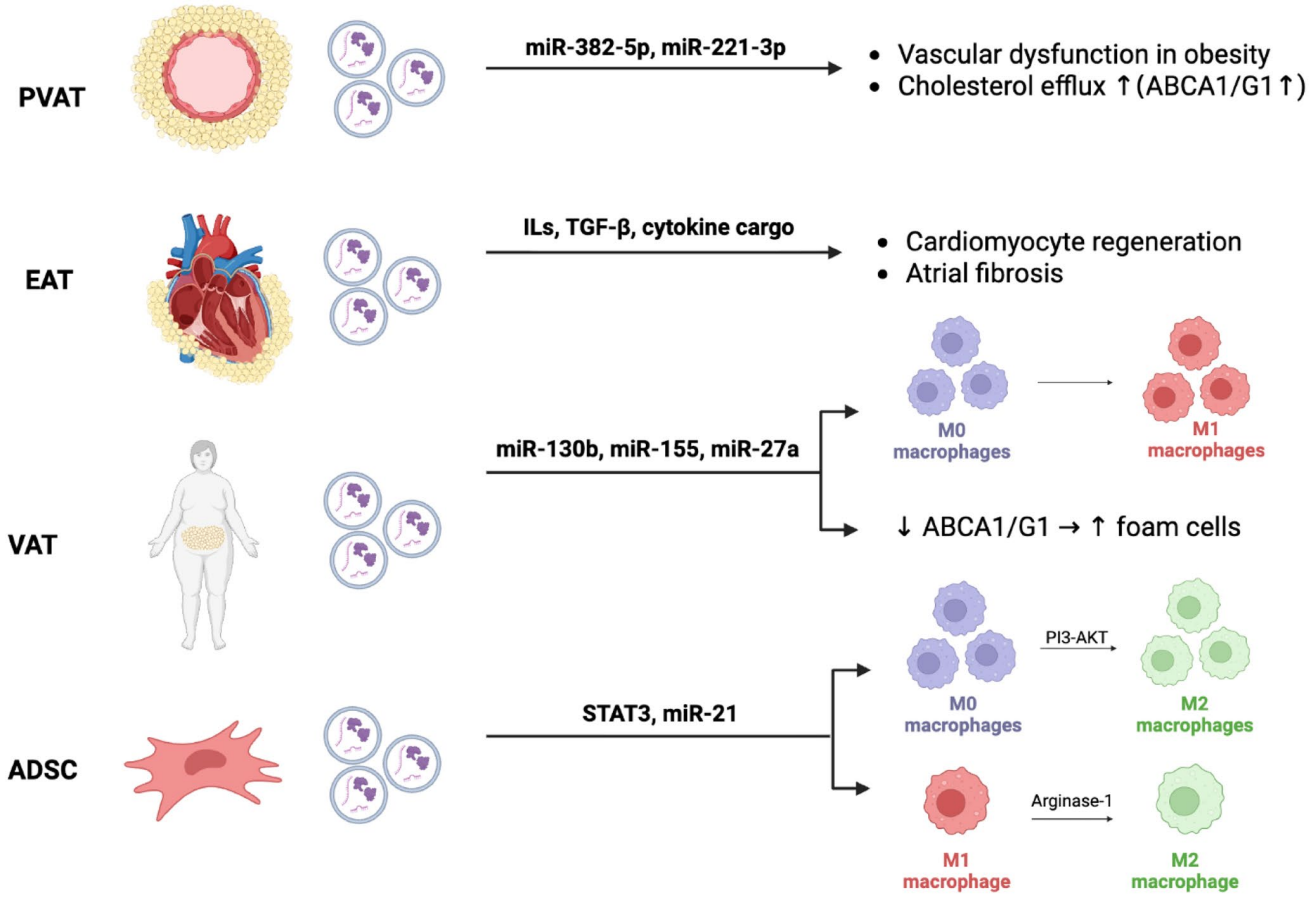
Different types of adipose‐derived extracellular vesicles (ADEVs). ABCA1/G1, ATP‐binding cassette transporter A1/G1; ADSC, adipose‐derived stem cells; AKT, protein kinase B; EAT, epicardial adipose tissue; PI3K, phosphoinositide 3‐kinase; PVAT, perivascular adipose tissue; VAT, visceral adipose tissue.

Atherosclerosis, the leading cause of cardiovascular mortality worldwide, is characterized by the pathological lipid accumulation within arterial walls and underlies severe conditions such as myocardial infarction, stroke, coronary artery disease (CAD), and peripheral artery disease [3, 43]. ADEVs are secreted by several adipose depots of particular relevance to CAD, including perivascular adipose tissue (PVAT), epicardial adipose tissue (EAT), and visceral adipose tissue (VAT) (Figure 1). Current evidence suggests that ADEVs exert protective effects against atherosclerosis through three primary mechanisms: modulation of macrophage foam cell formation, promotion of endothelial repair and vascular regeneration, and regulation of lipid metabolism [44, 45, 46].

### 3.1 Endothelial Protection and Vascular Regeneration

**FIGURE 2:**
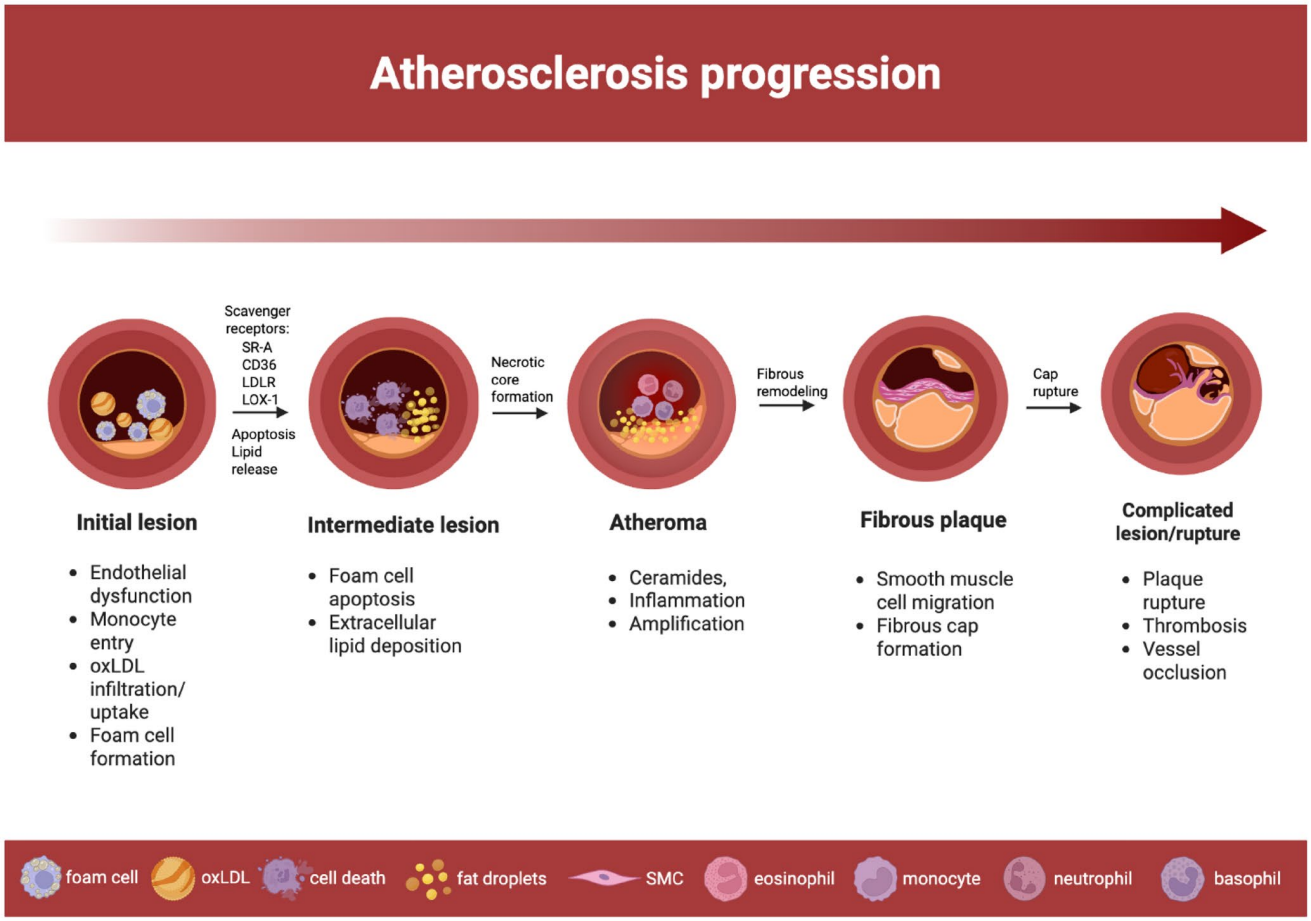
Stages of atherosclerosis pathogenesis. Sequential stages of atherosclerosis development are illustrated, from endothelial dysfunction and foam cell formation to necrotic core expansion, fibrous cap remodelling, and thrombotic occlusion following plaque rupture. Key molecular events include oxLDL uptake via scavenger receptors, impaired cholesterol efflux (downregulation of ABCA1/G1 expression), ceramide‐driven inflammation, and smooth muscle cell (SMC) mediated fibrous cap formation.

The pathogenesis of atherosclerosis can be divided into three phases: initiation, progression, and complications [43]. Endothelial dysfunction is a key trigger in the early stages of atherosclerotic plaque formation, leading to the activation of inflammatory cascades [47]. The ability of endothelial cells to adapt to disturbed blood flow, particularly at arterial branching sites and bifurcations, where atherosclerosis preferentially occurs, is crucial in atherosclerosis progression [39]. As plaques enlarge and become unstable, luminal narrowing may lead to ischemia, often requiring revascularization to restore perfusion [48]. Figure 2 illustrates the stages of atherosclerosis pathogenesis.

When guideline‐recommended therapeutic approaches for ischemic vascular diseases—revascularization or pharmacotherapy—fail to restore adequate blood flow, alternative strategies may be considered. Therapeutic angiogenesis, defined as the formation of novel blood vessels from pre‐existing endothelium, is a novel approach built on recent advances in molecular biology and holds promise in such cases [13, 48]. Ischemic injury initiates an inflammatory response that includes both pro‐inflammatory signalling required for tissue clearance and subsequent anti‐inflammatory mechanisms necessary for repair [49, 50]. ADEVs modulate this immunological response by influencing macrophage polarization. They deliver miR‐21, which activates the PI3K‐AKT pathway, and Colony Stimulating Factor‐1, promoting macrophage polarization from a proinflammatory M1 phenotype toward a reparative M2 phenotype, thereby supporting endothelial proliferation, migration, and angiogenesis. ADSC‐derived EVs also exhibit direct effects on the endothelial cells by delivering the glycoprotein stanniocalcin‐1 (STC‐1), which has been shown in vivo to promote re‐endothelialization following vascular injury [51].

### 3.2 Modulation of Macrophage Foam Cell Formation

Foam cell formation is a central process in atherosclerosis, resulting from an imbalance in cholesterol uptake and efflux within macrophages. Uptake of oxidized low‐density lipoprotein (ox‐LDL) via scavenger receptors such as SR‐A, CD36, LDLR, and LOX‐1, combined with impaired efflux via ATP‐binding cassette transporters (ABCA1, ABCG1), drives lipid accumulation [52, 53].

Current evidence suggests that EVs derived from PVAT modulate these processes. Several studies reveal that PVAT‐EVs exert atheroprotective effects by attenuation of lipid‐rich macrophage formation through mechanisms such as miR‐382‐5p‐mediated enhancement of cholesterol transport [54, 55]. More specifically, PVAT‐EVs have been reported to downregulate scavenger receptor‐A (SR‐A), thereby reducing ox‐LDL uptake, while upregulating the cholesterol efflux transporters ABCA1 and ABCG1, ultimately limiting foam cell formation [56]. Interestingly, EVs isolated from patients with CAD exhibit significantly lower levels of miR‐382‐5p compared with those from healthy individuals [55], suggesting impairment of this mechanism in this condition.

Similarly, ADSC‐EVs exert anti‐atherogenic effects by reprogramming macrophage polarization. In diet‐induced obese mice, ADSC‐EVs deliver active STAT3 to macrophages, which upregulates arginase‐1 and interleukin‐10 expression while suppressing pro‐inflammatory mediators. The STAT3‐Arg‐1 axis promotes M2 macrophage polarization, reducing vascular inflammation and enhancing lipid clearance. Additionally, M2 macrophages secrete catecholamines that stimulate the beigeing of white adipose tissue, a metabolic shift associated with increased energy expenditure and reduced lipid accumulation [57].

### 3.3 Regulation of Lipid Metabolism

Obesity and obesity‐induced inflammatory disease are well‐recognized contributors to the initiation and progression of atherosclerosis, largely through their effects on lipid metabolism [58]. ADEVs mediate these effects by transporting bioactive lipids that impair cholesterol efflux and promote inflammation. Under physiological conditions, ADEVs support lipid homeostasis; however, in obesity, their cargo shifts toward a pro‐atherogenic profile. Notably, obesity‐associated ADEVs are enriched in ceramides, which disrupt macrophage cholesterol homeostasis, facilitate foam cell formation, and accelerate plaque development [57, 59].

Hypoxia‐driven metabolic changes further exacerbate this effect. In obese adipose tissue, hypoxic conditions increase the expression of lipogenic enzymes such as acetyl‐CoA carboxylase, glucose‐6‐phosphate dehydrogenase, and fatty acid synthase, which are subsequently packaged into ADEVs [46]. These enzyme‐enriched vesicles contribute to systemic lipid dysregulation and may promote lipid accumulation in vascular cells, accelerating atherosclerosis development.

In addition to lipid alterations, obesity modifies the small RNA cargo of ADEVs. miRNAs such as miR‐155, miR‐27a, and miR‐130b, which impair cholesterol efflux pathways and promote foam cell formation, are enriched in obesity‐derived ADEVs [34, 35]. Furthermore, VAT‐derived EVs have a stronger pro‐atherogenic effect than those from subcutaneous fat, underscoring origin‐specific variation in EV cargo [60].

Finally, obesity not only alters the cargo of ADEVs but also increases their overall secretion, thereby amplifying lipid transport dysregulation and promoting macrophage lipid accumulation [61]. Collectively, these findings suggest that therapeutic strategies aimed at modulating ADEV lipid composition, cargo, or secretion have the potential to mitigate obesity‐driven atherosclerosis [62].

## 4 The Pro‐Regenerative Potential of Adipose‐Derived EVs in the Context of Myocardial Tissue

### 4.1 Cardioprotective Effects of ADEVs in Myocardial Tissue

As the body of evidence on the potential positive impact of ADEVs in CVD continues to grow, more attention is being paid to their protective value for myocardial tissue [63].

MI is an ischemic condition characterized by death of cardiomyocytes due to prolonged limited or blocked blood flow to a part of the heart. ADEVs have demonstrated cardioprotective effects in both in vitro and in vivo models. They reduce inflammatory response and cardiomyocyte apoptosis in vitro [37, 38, 64, 65]. In vivo studies have revealed reduced infarct size, decreased cardiomyocyte apoptosis, increased angiogenesis, decreased serum levels of MI biomarkers, and improved left ventricular ejection fraction [65, 66].

### 4.2 Mechanistic Insight

Various plausible mechanisms underlying the cardioprotective effect of ADEVs have been proposed, among which the most prominent are their anti‐apoptotic effect [67], promotion of angiogenesis [36], and immunomodulatory properties [68].

The mechanisms through which ADEVs exert these benefits in MI differ functionally from their roles in atherosclerosis, reflecting their involvement in post‐ischemic myocardium repair rather than plaque regulation.

#### 4.2.1 Anti‐Apoptotic Mechanisms

Hypoxia‐inducible factor‐1α (HIF‐1α) is a transcription factor whose expression is increased under hypoxic conditions, such as those seen in MI. It promotes cell survival by stimulating angiogenesis and, most prominently, by downregulating apoptosis [18]. ADEVs/ADSC‐EVs deliver miRNAs that act on hypoxia‐responsive pathways in MI. In particular, miR‐93‐5p–containing EVs attenuate myocardial injury and apoptosis in vivo [69], and miR‐31 alleviates MI via the FIH1/HIF‐1α axis [70]. Additional anti‐apoptotic effects have been reported for miR‐221 in ASCs‐EVs [66], and for miR‐214 transferred to cardiomyocytes after acute myocardial infarction (AMI) [67]. Hypoxia‐preconditioned MSC‐EVs also mitigate MI injury by modulating the TXNIP–HIF‐1α pathway [64].

Nuclear factor E2‐related factor 2 (Nrf2) is a molecule with cytoprotective properties, which is upregulated by miRNA‐24‐3p. A recent study by Huang et al. revealed this miRNA as a predominant therapeutic cargo in ADSC‐derived EVs [71].

#### 4.2.2 Promotion of Angiogenesis

In the myocardial setting, angiogenesis supports restoration of perfusion in ischemic tissue. ADEVs/ADSC‐EVs promote post‐infarction angiogenesis through delivery of pro‐angiogenic miRNAs, including miR‐126, miR‐31, and miR‐205, which enhance endothelial migration and proliferation [36, 37, 70, 72]. In vivo, administration of ADEV/ADSC‐EVs increases capillary density in the infarct border zone and improves tissue perfusion and ventricular function in MI models [65, 70, 72].

#### 4.2.3 Immunomodulatory Mechanisms

Following MI, ADEVs/ADSC‐EVs modulate inflammatory signalling in the myocardium in a miRNA‐dependent manner. In rodent and large‐animal models, EV‐based interventions reduce inflammatory injury and favour reparative responses, limiting adverse remodelling [38, 73]. Reviews focused on post‐MI EV therapy also highlight inflammation‐targeting as a key mode of action [68]. Given the current evidence base, this immunomodulatory activity is best attributed to EV‐mediated delivery of regulatory miRNAs in the infarct microenvironment, with specific pathways varying across studies [38, 68, 73].

### 4.3 Specific miRNAs and Their Molecular Targets

Adipose‐derived mesenchymal stem cells (ADMSC)‐derived miRNA‐671 has been shown to attenuate the TGFBR2/Smad2 axis [74]. This pathway is implicated in the activation of cardiac fibroblasts, which may contribute to cardiac fibrosis after MI [19].

Early growth response factor 1 (EGR1) causes myocardial damage following AMI by promoting various pathological processes, including inflammation, apoptosis, and ferroptosis [20, 21, 44]. Pan et al. showed that exosomes derived from miRNA‐146a‐overexpressing ADSC ameliorated inflammatory response, apoptosis induced by AMI, and cardiac fibrosis more effectively than unmodified‐ADSC‐derived exosomes, and miRNA‐146a‐EGR1 interaction was suggested as a plausible mechanism [38].

### 4.4 Unidentified Mediators and Signalling Pathways

Whereas in the studies mentioned above, specific miRNAs responsible for the protective effects of this pathway are believed to play a role in activating cardiac fibroblasts, which may lead to cardiac fibrosis after on myocardial tissue have been identified, some authors report the contents of ADEVs they believe contribute to these effects without identifying the underlying pathways (Table 1).

**TABLE 1:** Summary of miRNA present in ADEV, including type of cells used in the study, model system as well as plausible effect.

Identified miRNA	Targeted pathway	Source cell	Model system	Effect	References
miRNA‐31	FIH1/HIF‐1α	ADSCs	In vivo: mouse hindlimb ischemia and AMI models; In vitro: human microvascular endothelial cells	Endothelial cell migration and angiogenesis promotion, improvement of blood perfusion, infarct size reduction	[70]
miRNA‐224‐5p	TXNIP/HIF‐1α	Hypoxia‐preconditioned ADSCs	In vivo: C57BL/6 mouse model In vitro: cardiomyocytes of neonatal C57BL/6 mice	Reduction of infarct size and apoptotic degree, cardiac function improvement	[64]
miRNA‐93‐5p	TLR4/NF‐kB	ADSCs	In vivo: rat model of AMI In vitro: hypoxic H9c2 cells	Reduction of infarct volume, suppression on ischemia‐induced myocardial apoptosis	[69]
miRNA‐671	TGFBR2/Smad2	ADMSCs	In vivo: mouse AMI model In vitro: mouse cardiomyocytes	Enhanced viability, reduced apoptosis of cardiomyocytes, reduced myocardial fibrosis and inflammation	[74]
miRNA‐146a	EGR1/TLR4/NFκB	ADSCs	In vivo: rat model of AMI In vitro: hypoxic H9c2 cells	Reduction of AMI‐induced apoptosis, inhibition of inflammatory response, cardiac function improvement	[38]
miRNA‐24‐3p	Transcription factor Nrf2	ADSCs	In vivo: mouse myocardial ischemia–reperfusion model	Reduction of infarct volume, suppression on ischemia‐induced myocardial apoptosis	[71]
miRNA‐205	—	ADMSCs	In vivo: mouse AMI model In vitro: HMEC‐1 cells	Reduced myocardial fibrosis and cardiomyocytes apoptosis, angiogenesis promotion	[72]
miRNA‐126	—	ADSCs	In vivo: rat model of AMI In vitro: hypoxic H9c2 cells	Reduction of inflammation factor expression during hypoxia induction, decrease in the area of infarction of myocardium and angiogenesis promotion	[37]
miRNA‐221	—	ADSCs	In vivo: rat model of AMI In vitro: hypoxic H9c2 cells	Reduction of AMI‐induced apoptosis, lower serum troponin levels, cardiac function improvement	[66]

Abbreviations: ADMSCs, adipose‐derived mesenchymal stem cells; ADSCs, adipose‐derived stem cells; AMI, acute myocardial infarction.

The Wnt/β‐catenin pathway is known to regulate cardiomyocyte responses to injury [22]. Cui et al. suggested that activation of this signalling axis by exosomes secreted by ADMSC is a plausible explanation for their cardioprotective impact [65]. However, the specific microRNAs or other signalling factors responsible for this effect have not yet been identified, and the detailed mechanisms remain to be elucidated.

### 4.5 Non‐miRNA Cargo and Other Mechanisms of Cardioprotection

While most studies identify miRNAs as the principal ADEV cargo mediating cardioprotective effects, Crewe et al. reported that, under conditions of energetic stress such as obesity, adipocytes can release small EVs containing oxidatively damaged mitochondrial components, which are subsequently taken up by cardiomyocytes. This process induces a reactive oxygen species burst in the myocardium, preconditioning the tissue and protecting it from ischemic injury [23].

Huang et al. found that exosomes from sirtuin 1‐overexpressing ADSC‐EV promoted migration and tube formation of endothelial progenitor cells derived from patients after AMI by increasing the expression of C‐X‐C motif chemokine 12 (CXCL12) and Nrf2. Moreover, they delivered these particles into the myocardium of mice after AMI induction, resulting in reduced AMI‐induced inflammation [24]. EAT‐derived EVs promoted the expression of transcription factors specific for cardiomyocytes in cardiac fibroblasts under ischemic conditions, and downregulated both fibroblast and cardiac biomarkers (vimentin, FSP1, podoplanin, and troponin‐I, connexin‐43, respectively). LGALS1, PRDX2, and CCL2 were identified as key proteins involved in the regenerative process [75].

Whereas most studies have been conducted either in vitro or in small animals, Monguió‐Tortajada et al. used a porcine model of MI. They placed a decellularized pericardial scaffold enriched with peptide hydrogel and extracellular vesicles from porcine cardiac adipose tissue mesenchymal stromal cells over the infarcted myocardium. Compared with the control group, treated animals had higher right ventricular ejection fraction, less ventricular dilatation, and reduced scar size. Additionally, the treatment modulated both local and systemic inflammation [73].

## 5 Clinical Use of ADEVs in Therapies Targeting the Cardiovascular System

### 5.1 Description of the Main Trends

Clinical evidence directly evaluating ADEVs in therapies targeting specifically the cardiovascular system, which represents the primary focus of this review, has not been documented to date. Administration of isolated ADEVs to benefit from their immunomodulatory and anti‐inflammatory properties has been clinically introduced in the context of psoriasis, Alzheimer disease, severe COVID‐19, and wound healing [76, 77, 78]. Accordingly, clinical studies employing ADSCs are discussed in this section, as ADEVs are regarded as principal mediators of the paracrine mechanisms underlying ADSCs‐based therapeutic effects [79].

Despite promising preclinical results demonstrating the cardioprotective properties of ADEVs in‐based therapies, translation into clinical settings remains challenging. As of March 2026, no stem cell‐based products have received regulatory approval for clinical use in cardiological indications. At present, this approach is being examined in Phase 1 or Phase 2 clinical trials, primarily for patients with refractory chronic myocardial ischemia with left ventricular dysfunction, advanced ischemic heart failure, or ischemic cardiomyopathy who are unsuitable for surgical revascularization. Projects investigating ADSCs in this area remain scarce. Notably, many registered trials have been discontinued, while the results of others have not been published (Table 2).

**TABLE 2:** Registered clinical trials exploring cardioprotective properties of adipose‐derived stem cells.

Trial	Principal investigator	Location	Status	Phase	Start date	Completion date	Citations
NCT00426868 (PRECISE)	E. C. Perin	Denmark, Netherlands, Spain	Completed	1	2007‐01	2012‐03	[30, 80]
NCT00442806 (APOLLO)	J. H. Houtgraaf	Netherlands, Spain	Completed	1	2007‐11	2012‐04	[29, 81]
NCT01449032 (MyStromalCell)	A. A. Qayyum	Denmark	Completed	2	2010‐04	2014‐01	[82]
NCT01473433 (AdiFlap)	A. Bayes‐Genis	Spain	Completed	1, 2	2012‐01	2014‐12	[83]
NCT01556022 (ATHENA I)	T. D. Henry	United States	Completed	2	2012‐06	2016‐10	[31, 84]
NCT01709279	NA	Japan	Unknown (no updates since 2017‐08)	NA	2012‐08	2019‐08	[85]
NCT01216995 (ADVANCE)	S. Kesten	Netherlands, Poland	Completed without publication of the results	2	2012‐09	2014‐05	[86]
NCT02052427 (ATHENA II)	T. D. Henry	United States	Completed	2	2014‐01	2016‐10	[31, 87]
NCT01974128 (Acute MI)	NA	United States	Withdrawn (no updates since 2017‐07)	NA	2014‐10	2017‐07	[88]
NCT02387723 (CSCC_ASCII)	A. A. Qayyum	Denmark	Completed	1	2014‐12	2015‐11	[89, 90]
NCT03092284 (CSCC_ASCII)	A. A. Qayyum	Denmark	Completed	2	2015‐09	2021‐07	[89, 91]
NCT03272191	S. McQuillan	United States	Withdrawn (no updates since 2017‐09)	NA	2016‐09	2018‐12	[92]
NCT02673164 (SCIENCE)	A. A. Qayyum	Denmark, Germany, The Netherlands, Austria, Slovenia, and Poland	Completed	2	2017‐01	2020‐12	[93]
NCT04695522	T. Kawamura	Japan	Completed	1	2021‐04	2021‐10	[94]
NCT04005989 (ADMIRE)	NA	Brazil	Withdrawn (no updates since 2022‐09)	3	2021‐12	2022‐12	[95]
NCT06705023	A. Baigenzhin	Kazakhstan	Recruiting	2	2024‐11	2025‐12	[96]
NCT06840275 (ARIISE)	A. A. Qayyum	Denmark	Not yet recruiting	2	2025‐09	2028‐09	[97]

The translation of ADEV‐based therapies from research to clinical practice is hindered not only by substantial heterogeneity in EVs isolation and characterization methods, but also by considerable variability in study design, including cell sources, administration routes, and dosing regimens [98]. Moreover, comparability across trials is further limited by small sample sizes and inconsistent endpoints. Table 2 provides a summary of all trials investigating ADSCs in cardiovascular diseases registered on clinicaltrials.gov, while Table 3 summarizes the results of published trials.

**TABLE 3:** Completed and future clinical trials exploring cardioprotective properties of adipose‐derived stem cells.

Trial	Status	Phase	Indication	Number of patients (placebo/treatment)	Intervention	Cells	Dosing	Feasibility endpoint measure	Follow‐up	Conclusion	Citations
NCT00426868 (PRECISE)	Completed	1	Ischemic cardiomyopathy	6/21	Direct injection of ADRCs into the ischemic region of Left Ventricle using NOGA XP system	Freshly isolated autologous adipocytes harvested in liposuction	0.4 × 10^6^ cells/kg, 0.8 × 10^6^ cells/kg	LVEF, WMIS, LVESV, LVEDV total and infarcted left ventricular masses, METs, VO_2_max	36 months	Therapy is safe and feasible, may preserve ventricular function, myocardial perfusion and exercise capacity	[30, 80]
NCT00442806 (APOLLO)	Completed	1	STEMI	4/10	Intracoronary infusion of ADRCs within 24 h after successful primary percutaneous coronary intervention	Freshly isolated autologous adipocytes harvested in liposuction	20 × 10^6^ cells	LVEF, infarct size in LGE MRI, SPECT	6 months	Therapy is safe and feasible, improves cardiac function, perfusion and reduces myocardial scar formation	[29, 81]
NCT01449032 (MyStromalCell)	Completed	2	Chronic ischemic heart disease and refractory angina	20/41	Direct injection of ADRCs using MYOSTAR injection catheter into the ischemic region of Left Ventricle guided by NOGA system, 10–15 injections of 0.2 mL	VEGF‐A165‐ stimulated autologous ADSCs, obtained from abdominal liposuction and cultured for 7 days	Total amount of cells reached after 2 passages.	Bicycle exercise test, METs, exercise performance measured in watts, CCS and NYHA class, weekly frequency of angina attacks	36 months	Therapy improved cardiac symptoms, but exercise capacity remained unchanged	[82]
NCT01473433 (AdiFlap)	Completed	1, 2	Chronic transmural myocardial scar	4/5	Adipose Graft Transposition Procedure performed on non‐revascularizable area	Autologous pericardial adipose graft	to ensure full coverage of the necrotic zone by the flap	LVEF, CO, SV, EDWM, LVEDV, LVESV in MRI, NTproBNP, troponin I, NYHA class, Framingham derived clinical	12 months	Therapy is safe, but it does not improve left ventricular function. It may be efficacious in some groups of patients—with the largest necrotic area, where significant improvements have been observed	[83]
NCT01556022 (ATHENA I)	Completed	2	Refractory Chronic Myocardial Ischemia with Left Ventricular Dysfunction	14/28^*^	Direct injection of ADRCs using MYOSTAR injection catheter into the ischemic region of Left Ventricle guided by NOGA system, 15 injections of 0.2 mL	Fresh autologous ADSCs, obtained from abdominal liposuction	0.4 × 10^6^ cells/kg body weight, not to exceed 40 × 10^6^ cells	VO_2_max, MLHFQ, symptom‐limited exercise treadmill testing using a modified Naughton protocol, LVESV, LVEDV, SF‐36, CCS and NYHA class	6 months	Therapy is feasible, improves VO_2_ max, NYHA and CCS class. It does not improve left ventricular function nor volumes. Authors suggest a benefit in “no option” CAD patients.	[31, 84, 87]
NCT02052427 (ATHENA II)	Completed	2	Refractory Chronic Myocardial Ischemia with left ventricular dysfunction	14/3^*^			0.8 × 10^6^ cells/kg body weight, not to exceed 80 × 10^6^ cells				
NCT02387723 (CSCC_ASCII)	Completed	1	Symptomatic chronic ischemic Heart failure with reduced LVEF	NA	Direct injection of ADRCs using MYOSTAR injection catheter into the ischemic region of Left Ventricle guided by NOGA system; 12–15 injections of 0.3 mL solution except the first one of 0.4 mL	Cryopreserved allogeneic ADSC product (CSCC_ASC)	100 × 10^6^ cells	LVESV, LVEDV, in ECHO, NYHA class, 6MWT, KCCQ, CK‐MB, CRP	6 months	Therapy is safe but does not improve clinical symptoms nor myocardial function or structure	[89, 90, 91]
NCT03092284 (CSCC_ASCII)	Completed	2		27/54							
NCT02673164 (SCIENCE)	Completed	2	Symptomatic chronic ischemic heart failure with reduced LVEF	43/90	Direct injection of ADRCs using MYOSTAR injection catheter into the ischemic region of left ventricle guided by NOGA system; 15 injections of 0.3 mL solution	Cryopreserved allogeneic ADSC product (CSCC_ASC)	100 × 10^6^ cells	LVESV, LVEDV, LVEF, 6MWT, NYHA class, KCCQ, EQ‐5D‐3L, 6MWT, NT‐proBNP, CRP	12 months	Therapy is safe but does not improve left ventricular function nor the quality of life	[93]
NCT04695522	Completed	1	Ischemic cardiomyopathy with reduced LVEF	3/3	Perioperative cell spray transplantation of ADSCs during CABG	Cryopreserved allogeneic ADSC product (ADR‐002K)	300 × 10^6^ cells	LGE MRI, LVEF, LVEDV, LVESV, 6MWT, MLHFQ, NYHA class	24 weeks	Therapy is safe and can enhance cardiac function	[94]
NCT06705023	Recruiting	2	Chronic congestive heart failure with reduced LVEF	24/12	Retrograde intra‐cardiac venous injection of UA‐ADRCs	Fresh autologous, ADSC isolated from lipoaspirate	Unknown	NT‐proBNP, 6MWT, MLHFQ, cMRI (relative amount of left ventricular scar tissue, left ventricular bull's eye segmental contracting areas)	6 months	NA	[96]
NCT06840275 (ARIISE)	Not yet recruiting	2	Recently Diagnosed Non‐ischemic Heart Failure	Estimated 90	Two intravenous infusions of ADSCs 4 weeks apart	Allogeneic MSCs obtained from adipose tissue (C2C_ASC110)	110 × 10^6^ cells	LVEF, LVESV, LVEDV, KCCQ, EQ5D5L, 6MWT, Pro‐BNP	12 months	NA	[97]

Abbreviations: 6MWT, 6‐minute walk test; ADRC, adipose‐derived regenerative cells; CABG, coronary artery bypass graft; CCS, Canadian Cardiovascular Society; CO, cardiac output; EQ‐5D‐3L, EuroQol Five Dimensions questionnaire, Three Levels; KCCQ, Kansas City Cardiomyopathy Questionnaire; LGE MRI, late gadolinium enhancement magnetic resonance imaging; LVEDV, left ventricular end‐diastolic volume; LVEF, left ventricular ejection fraction; LVESV, left ventricular end‐systolic volume; MET, metabolic equivalent; MLHFQ, Minnesota Living with Heart Failure Questionnaire; MSC, mesenhymal stem cells; NYHA, New York Heart Association; SPECT, single photon emission computed tomography; STEMI, ST‐elevation myocardial infarction; UA‐ADRC, uncultured autologous adipose‐derived regenerative cells; WMIS, wall motion score index.

^*^ Due to the low number of patients recruited for the ATHENA II trial, both treatment groups were merged. The control group consisted of 14 patients.

#### 5.1.1 Cell Harvesting Approaches and Delivery Routes

Initially, all reported trials employed autologous cells freshly harvested during abdominal liposuction [29, 30, 31]. The MyStromalCell was a pioneer trial that used autologous cells cultured ex vivo [99]. A more recent approach was proposed by the developers of the CSCC_ASC product, who established a manufacturing protocol for clinical‐grade cryopreserved allogeneic ADSCs derived from healthy donors and successfully completed two clinical trials evaluating their application [89]. The rationale for this innovation was to obtain a more homogeneous, ready‐to‐administer therapeutic product that facilitates rapid, straightforward application in clinical settings. However, evidence of clinical benefits for this intervention is lacking.

This concept was further explored by Kawamura et al., who in 2024 published positive phase 1 results of a trial investigating the application of an allogeneic cryopreserved ADSC‐based product, ADR‐002 K, delivered in an innovative “cell spray” form [25].

Numerous studies have investigated the direct injection of ADSCs into the ischemic region of the left ventricle using the MYOSTAR injection catheter, guided by the NOGA system (Biologics Delivery System, CA, US) [30, 31, 89, 99]. Intracoronary delivery of cells, in the form of an adipose tissue flap graft, is another recently employed strategy [29, 100]. The novel perioperative “cell spray therapy” has also garnered interest among investigators [25]. One approach, which improved left ventricular function in all treated patients (*n* = 3), involved mixing cells with fibrin glue and spraying the mixture onto the heart surface during coronary artery bypass grafting (CABG). These findings, although promising, should be regarded as preliminary until confirmed in larger patient cohorts [25].

#### 5.1.2 Target Populations

Another significant limitation in the development of ADSC‐based clinical trials is the limited number of recruited patients, which in some studies has not exceeded 10 per study arm [29, 100]. Based on these rationales, two separate ATHENA trials had to be merged into a single treatment group, precluding independent evaluation of the effects of different dosing regimens [31].

### 5.2 Results

Completed clinical trials to date have established a solid foundation for further investigation, as all have confirmed the safety of adipose‐derived mesenchymal tissue transplantation. However, given the limited sample sizes, these findings should be considered hypothesis‐generating rather than conclusive, pending validation in larger, adequately powered trials. Importantly, evidence regarding efficacy remains inconsistent. Only a small number of studies have demonstrated significant clinical benefit, such as improved left ventricular ejection fraction (EF) in patients with ST‐segment elevation MI [29], ischemic heart failure with reduced left ventricular EF [101], or ischemic cardiomyopathy with reduced EF [25]. In another trial involving patients with ischemic cardiomyopathy, ventricular function was preserved in the treated group, while in the placebo‐controlled group, a decrease of this parameter was observed [30]. Further clinical benefits of ADSCs in chronic myocardial ischemia include reduction in left ventricular necrotic mass and improvements in left ventricular end‐diastolic volume (LVEDV), although these effects were observed in patients with the highest disease severity [100]. Patient‐reported outcomes assessed by Minnesota Living with Heart Failure Questionnaire (MLHFQ) and The Short Form Health Survey (SF36) scores [31] revealed improvement in cardiac symptoms and a reduction of weekly angina attacks [99]. Conversely, in many patients with symptomatic chronic ischemic heart failure and reduced LVEF, ADSC therapy did not produce significant symptomatic relief [89]. In the MyStromalCell study, the intervention led only to an improvement in cardiac symptoms, with exercise capacity remaining unchanged, whereas both parameters deteriorated in the placebo group [99]. In a relatively large cohort of 30 patients with ischemic heart failure and reduced left ventricular EF, who were ineligible for PCI or CABG, stroke volume increased one year after injection of ADSCs. However, no significant changes in LVEF or cardiac output were observed [101].

Registered clinical trials exploring the cardioprotective effects of ADSCs are presented in Table 3. Completed clinical trials investigating the cardioprotective properties of ADSCs are summarized in Table 3.

## 6 Safety Issues of ADEVs‐Based Therapies

Numerous studies highlight the regenerative and anti‐atherosclerotic actions of ADEVs, but it is also important to acknowledge their potential harmful effects. A meta‐analysis focused on the safety aspect of ADSCs‐based clinical trials indicates a favourable safety profile in patients with ischemic heart disease, with no severe adverse events reported, supporting the overall tolerability of adipose‐derived cell therapies in clinical settings [102]. Even though further verification of their safety, as well as potential complications mechanisms, should not be withdrawn. ADEVs are highly context‐dependent, and under pathological conditions, they may contribute to disease progression. For example, vesicles secreted from adipose tissue associated with tumours can facilitate cancer cell proliferation, migration, and metastasis by transferring oncogenic proteins, lipids, or miRNAs [26, 103, 104]. Similarly, ADEVs derived from obese or inflamed adipose tissue have been reported to promote vascular inflammation and endothelial dysfunction, thereby exacerbating cardiometabolic risk [105, 106]. EAT‐derived EVs contribute to atrial fibrosis through inflammatory cytokine cargo in atrial fibrillation [107]. These findings emphasize that the cargo of ADEVs is not universally protective but is shaped by the metabolic and pathological state of their parent cells.

ADEVs, bioactive molecules that carry numerous proteins and lipids on their surface, exhibit natural immunogenicity, which should also be considered a potential safety issue. Various administration routes of EVs, as well as dosage, play a crucial role in this process as they result in heterogeneous tissue distribution and engagement with distinct components of the immune system. Moreover, off‐target effects of ADEVs remain a potential concern, as EVs may accumulate in non‐cardiac tissues such as the liver, lungs, and spleen, potentially interacting with unintended immune or parenchymal cells [108, 109]. Results of human EVs administration in non‐human primates suggest that higher or repeated exposures can accelerate clearance, enhance lysosomal activity, and trigger immune recognition [110]. In a mouse model, ADEVs were administered at doses corresponding to the human equivalent of up to 5.8 × 10^10^ particles per kg of body weight and did not induce acute or subchronic toxicity, providing an important reference for future human studies [111].

Future translational efforts should therefore carefully consider donor characteristics and standardize isolation methods, dosing, and biodistribution to minimize the risk of transferring harmful signals.

## 7 ADEVs As a Potential Drug‐Delivery Platform

Whereas the primary focus in ADEVs‐related research in the cardiovascular system is on the potential therapeutic effects of their native content, a new potential application of these particles has emerged: as a drug delivery platform. Since transport of bioactive molecules is a major function of ADEVs in the body, they have a relatively high loading capacity [112]. Engineering strategies, such as surface functionalization, controlled cargo loading, and combination with biomaterials, can further enhance cardiac homing and on‐target efficacy [113].

Despite numerous recent studies using animal models and advances in engineering, this approach still faces many technological limitations. Concerns include a lack of tissue specificity and the absence of a less invasive method of administration than direct injection into the myocardium or pericardium [114].

## 8 Conclusions and New Directions

Recent studies on ADEVs have provided a deeper understanding of their properties and roles in both physiological and pathological processes. Moreover, advances in technology have enabled more detailed investigations of their biological content, offering new insights into the mechanisms underlying their actions.

Numerous studies support the significant role of ADEVs in both atherosclerosis and myocardial tissue regeneration following ischemia. In atherosclerosis, they influence macrophage foam cell formation, protect the endothelium, promote vascular regeneration, and regulate lipid metabolism. In the case of myocardial ischemia, ADEVs reduce the area affected by apoptosis and fibrosis, stimulate angiogenesis, and modulate inflammatory response.

With the expanding scope of knowledge in the field of ADEVs, new questions and challenges have emerged:

1. Unknown secretion patterns of ADEVs across various cardiovascular diseases.
2. Lack of clarity regarding the tissue‐specific origins of ADEVs carrying specific molecular cargo.
3. Inconclusive results of current studies due to small sample size.
4. Variability in the delivery protocols and dosage of ADEVs across studies.

Hopefully, upcoming studies will provide answers to at least some of these questions. The Cardiomesh II trial will evaluate the safety and efficacy of applying a membrane containing allogenic ADSCs during CABG to improve ventricular function in patients at high risk of developing heart failure [115]. Another double‐blinded trial in Denmark aims to test the safety and effect of AMSCs on cardiac function in patients with non‐ischemic heart failure [116]. Given their inherent tissue‐homing capacity and ability to carry bioactive cargo, ADEVs offer promising opportunities to explore alternative administration routes and optimize targeted delivery in cardiovascular therapies [112]. Various innovative smart materials and devices for enhanced EVs delivery have been under investigation in recent years [117, 118, 119]. Notably, many of them involve EVs derived from tissues other than adipocytes or have only been described in contexts other than cardiovascular diseases. A promising idea brought up by Hu et al. is the use of transdermal microneedle patches, which should sustain constant release of small EVs isolated from umbilical cord plasma into the circulation [120]. Moreover, non‐invasive routes of administration, including inhalation, have also been under investigation, such as inhalable stem cell exosomes to promote heart repair after myocardial infarction [121]. A different perspective was presented by Liu et al. in a mouse model, where inhalable CD34‐CD42b platelet‐targeting bispecific antibodies redirected stem cells from the lungs to repair heart injury [122]. All these approaches provide direction for further research, with a focus on ADEVs therapies in cardiovascular diseases.

## Author Contributions

K.S., W.O. and M.T. conceptualized the review and critically revised the manuscript, K.I., P.O. and W.P. conducted literature review and drafted the initial manuscript, K.I., P.O., D.T., and W.P. created tables and figures. All authors reviewed and approved the final manuscript.

## Funding

The authors have nothing to report.

## Ethics Statement

The authors have nothing to report.

## Consent

The authors have nothing to report.

## Conflicts of Interest

The authors declare no conflicts of interest.

## Data Availability

Data sharing not applicable to this article as no datasets were generated or analysed during the current study.
